# Patient perspectives of transitioning from prescription opioids to heroin and the role of route of administration

**DOI:** 10.1186/s13011-017-0137-y

**Published:** 2018-01-29

**Authors:** Laura B. Monico, Shannon Gwin Mitchell

**Affiliations:** Friends Research Institute, 1040 Park Avenue, Suite 103, Baltimore, MD 21201 USA

**Keywords:** Opioid use disorder, Opioid treatment, Prescription opioids, Heroin, Transition

## Abstract

**Background:**

As the availability of prescription opioids decreases and the availability of heroin increases, some prescription opioid users are transitioning to heroin. This study seeks to explore factors associated with respondents’ transition from prescription opioid use to heroin.

**Methods:**

In-depth, semi-structured qualitative interviews (*n* = 20) were conducted with buprenorphine patients in an opioid treatment program. Respondents were predominantly White (*n* = 13) and male (*n* = 13), with a range of treatment tenure (4 days to 2 years).

**Results:**

A vast majority of respondents in this study (*n* = 15) initiated opioid use with either licit (*n* = 8) or illicit (*n* = 7) prescription opioids (e.g. hydrocodone, oxycodone, morphine). Of these respondents, all but two transitioned from prescription opioids to heroin (*n* = 13). For those respondents who transitioned to heroin, most initiated heroin use intranasally (*n* = 12), after using prescription opioids in the same manner (*n* = 9), but before using heroin intravenously (*n* = 9). Respondents attributed this transition between substances to common explanations, such as “it’s cheaper” and “the same thing as pills.” However, respondents also dispel these myths by describing: 1) heroin quality is always uncertain, often resulting in spending more money over time; 2) dramatic increases in tolerance, resulting in spending more money over time and transitioning to intravenous use; 3) more severe withdrawal symptoms, especially when respondents transitioned to intravenous use.

**Conclusions:**

Understanding how route of administration and common myths shape key transition points for opioid users will allow practitioners to develop effective harm reduction and prevention materials that target individuals already using prescription opioids.

## Background

With greater than 10 million individuals in the US reporting non-medical use of prescription opioids (NMPOs) in 2014 [1], concern is still high regarding its association with high rates of morbidity and mortality [2]. Between 2000 and 2014, overdose rates attributable to NMPOs quadrupled [3], but simultaneously the US also experienced an increase in the rate of heroin use among both injection and non-injection users [4]. Not surprisingly, overdose rates attributable to heroin use also skyrocketed during these years [5], causing many researchers and public health officials to wonder if there was an association between the two phenomena [3].

Research during the last decade has established that individuals who abuse prescription opioids, especially those with a physiological dependence, may shift to heroin use, particularly when they already inhale or inject prescription opioids [6–17]. Several of these studies have found remarkably high likelihoods of heroin abuse after NMPO than without NMPO, as high as 19 times using data from 2011 [12], to nearly 40 times using data from 2013 [18].

Qualitative respondents in some of these studies have reported that the greater accessibility of heroin, and the relatively low cost compared to prescription opioids (because of their scarcity), facilitated their initiation to heroin use [6, 10, 16, 17]. Evidence such as this has lead several research studies to track participants’ trajectories of transition from NMPO to heroin [6–8, 15, 19]. However, these studies have indicated that only a fraction, generally less than 5%, of NMPO users transition to heroin at any point [12, 18]. From these studies we can conclude that NMPO is perhaps a widely accepted risk factor of later heroin use, with a majority of current heroin users reporting previous NMPO, but the transition to heroin from NMPO is rare, and generally occurs at a very low rate [3]. Nevertheless, heroin use and intravenous drug users are at much more serious risk for overdose, HIV, and other health-related complications, so it is important to identify patterns and risk factors that contribute to NMPO users’ transition to heroin and other routes of administration. This analysis sought to explore factors associated with respondents’ transition from prescription opioid use to heroin, using qualitative interview data from participants seeking buprenorphine treatment for opioid use disorder.

## Methods

In-depth, semi-structured interviews (*n* = 20) were conducted with buprenorphine patients in an opioid treatment program in Delaware. Participants were recruited to ensure demographic and experiential diversity, with specific attention paid to recruiting participants along racial, ethnic, and gender subsamples that roughly mirrored those of the larger clinic population. Although 20 interviews were completed, only those respondents who reported initiating their opioid use with prescription opioids were included in this analysis (*n* = 15). Overall, these respondents were predominately white (*n* = 9) and male (*n* = 10), with only a few identifying as Hispanic (*n* = 3). The age of respondents ranged from 23 to 60, with an average age of 36. Respondents provided informed consent, and all research activities were approved by the University of Delaware Institutional Review Board.

Qualitative interviews were completed over the course of 6 months during the winter of 2014–2015. Respondents were recruited from a single opioid treatment program that offered buprenorphine, along with psychosocial counseling elements. Participants were recruited during regular dosing hours, and interviews were completed on site in a private office area designated for research use.

Interviews varied in length (27 to 132 min), but averaged approximately 45 min. Interviews explored respondents’ substance use histories; treatment history and experience; issues encountered accessing buprenorphine treatment; family, friends’, and employer attitudes toward buprenorphine; criminal justice issues related to buprenorphine; plans to taper off buprenorphine; and, advice for future buprenorphine patients.

Qualitative data collection and analysis occurred simultaneously, allowing previously collected data to inform ongoing data collection and ensure saturation. Interviews were audio recorded and carefully transcribed to ensure accuracy. Following transcription, all participant names were replaced with pseudonyms. Interview transcripts were analyzed using an inductive coding scheme approach with ATLAS.ti qualitative analysis software. A primary coding scheme was developed based on the conceptual and thematic areas targeted in the interview guide. Within these primary level codes, the researchers inductively developed secondary level codes that were dependent upon narratives of the respondents. While the larger focus of the interviews was on buprenorphine respondents’ experiences in a traditional opioid treatment program that largely served methadone patients, the data presented here emerged organically when respondents’ substance use history was systematically analyzed.

## Results

The respondents in this study followed similar opioid use trajectories. As shown in Table 1, of the 20 respondents interviewed, 15 initiated opioid use with prescription opioids, about half of whom (*n* = 8) received their first opioid through a licit prescription for an illness or injury. Of the 15 respondents who initiated opioid use with prescription opioids, all but two eventually began using heroin. However, since the observation time between participants’ initiation of opioid use and the time of the interview were different, caution should be used when interpreting the significance of these two cases. Nine of the 13 respondents who eventually moved to heroin were using intravenously at the time of treatment entry. It should be noted, however, that none of these 15 respondents ever, at any point, injected prescription opioids during their years of opioid use—only heroin.

**Table 1:** Respondent characteristics who initiated opioid use with prescription opioids

Pseudonyms	Age	Years since first use of Rx opioid	Initiated w/legal rx	Route of Administration				
				Prescription Opioids			Heroin	
				Oral	Nasal	Injecting	Nasal	Injecting
Tim	36	13	N	x	x		x	x
Santiago	23	7	N		x		x	
Vince	28	4	N	x				
Mindy	32	7	Y	x	x		x	x
Luke	25	5	N		x		x	
Brian	31	12	N	x	x		x	x
Julie	32	13	Y	x	x		x	x
Matt	33	10	Y	x	x		x	x
Herc	33	8	Y	x				
Eric	35	2	N		x		x	x
Calvin	41	9	Y	x			x	
Shelby	55	16	Y	x				x
Tyra	30	6	N		x		x	x
Tami	47	15	Y	x			x	x
Carlotta	60	10	Y	x			x	

The respondents who initiated opioid use with prescription pain relievers had a history of opioid use ranging from 2 to 16 years ($\overline{X}$ = 8.8 years). All but one of the 15 respondents initiated prescription opioid use before the national policy and medical shifts of 2010–2011 began to occur.

### Transitioning to heroin and intravenous use

The most pervasive explanation people in this study gave for transitioning from prescription opioids to heroin concerned the cost of heroin relative to prescription opioids. Respondents reported that as the availability of, and access to, prescription opioid pills decreased over the few years prior to this research project, the price of the existing supply of these diverted pills increased. At the same time, the state of Delaware experienced a surge in the powdered heroin supply, thereby decreasing its price on the street.

When asked about how he generally chose to use prescription opioids, Brian (31/Male) responded:

*Yea, I just pop em, but then after awhile, sometimes I snort em, but it was never really my thing. And after awhile I just started dope, cause I mean someone says it’s cheaper, and I was like “ahhh…” it’s cheaper. Know what I mean? This will get you higher than that. You do two bags and it’s like taking a 30 [mg dose of oxycodone]. Know what I mean? And go from there. And here we are.*

Brian, who had been using prescription opioids pills for nearly 15 years, was not able to continue purchasing them as the cost rose and the availability declined. Once someone in his social network mentioned to him that heroin was less expensive, would actually get him higher than pills, and could easily replace the oxycodone he normally purchased, he decided to try it.

Luke (25/Male) was similarly influenced by someone in his substance user network who informed him that heroin was less expensive than the oxycodone he was using. When Luke first began taking 30 mg pills (often referred to as “Roxy’s” for Roxicodone), he would break them into pieces and use quarters of the single pill throughout the day. As his tolerance grew and his addiction intensified, he began to use more of the pill, more often during the day, until he was using multiple pills per day. As Luke used more pills, he had difficulty keeping up with the cost, so he decided to try heroin when an acquaintance told him it was cheaper.

Even though she had been using prescription opioids for 11 years at the time of her transition to heroin, Tami (47/Female) described her transition to heroin use in the following manner, also predicated upon cost.

*I was scared to death because I kept hearing people overdosing, and I said I would never do that, never, never, never, but there I was. My girlfriend, she’s like, “You’re paying $20 for a Perc 30, common, $20 you can get 6 bags of dope, they’re $5 a damn piece, and that $5 bag equals the $20 you’re paying for the Perc 30. That one bag equals that one pill.” And that’s what changed my mind. It tempted me, so I sniffed the one bag and within a half hour I was feeling good, I was able to move around, I was able to do my chores, whatever.*

The phenomenon of prescription opioid users transitioning to heroin due to the scarcity and rising cost of opioid pills is well-documented in the literature [6, 10, 16, 17]. However, one of the more interesting findings that emerged from these narratives is that respondents realized that while heroin may have been less expensive to purchase compared to a single 30 mg oxycodone pill, maintaining a heroin habit was ultimately costlier, even over a short period. Calvin (41/Male) described his experiences with buying heroin, which he approached with considerable thought and careful calculation.

***Respondent:*** *… a couple friends of mine saying that that’s just a powerful form of a Percocet. And I took a chance to see if that was the true results and effect of it, and you know, it’s an opiate, I mean heron is an opiate, it just that you don’t always know what you gonna get, versus the Percocet in the pill form that you get in a 10, a 15, or a 30, versus going to buy the drug on the street and you don’t know what it is.*

***Interviewer:*** *Yea, how much is going to be in there*

***Respondent:*** Just the quality of it, it could be a whole bunch of it, an ocean of it *Laughter* but the quality of it is just to keep the chase going. And it just starts off, like it’s all about who you know and never going in the streets, versus the price of any and everything. So whether you’re spending $50 or $25 for whatever the bundle or not, but if it ain’t good you’re going to be spending another $50 or $25. And this is all in one day. So then you re-evaluate your day, and be like, “damn, I did like 3 bundles and everything is garbage and I’m out of $150!”

When he purchased prescription opioids in the past, Calvin did not have to consider which dealers he was approaching, or how the cost of his habit would fluctuate on a daily basis. He knew exactly how many milligrams of oxycodone he needed to take each day to avoid being sick, and how much that dosage cost. Because of the tremendous variability in purity from one bag of heroin to another, respondents were never sure how many bags of heroin they were actually going to need each day to satiate their level of dependence. This unknown variable resulted in some respondents spending even more money to maintain their drug habits once they transitioned to heroin.

Santiago (23/Male), who began his opioid use by abusing prescription pills, generally maintained his use with two 30 mg oxycodone per day. When a friend mentioned to him that he could buy almost 20 bags of heroin for the same $50 he usually spent purchasing two pills per day, he decided to try it. After several months of heroin use, he realized that his tolerance was increasing; by the time he decided to enter buprenorphine treatment, he had been spending $75–$100 per day. Eric (35/Male) came to the same realization; that over time, heroin was “actually more expensive.” Although the transition to heroin from prescription pills initially supported the myth that heroin was cheaper, both Santiago and Eric realized over time that they needed to purchase more heroin due to an increase in their tolerance to opioids, which led both respondents to spend more money each day. In retrospect, Calvin, Santiago, and Eric were all able to come to the realization that the idea that heroin was cheaper than prescription pill is a myth that was debunked quickly after they had already transitioned to using heroin exclusively.

#### Route of administration and transitioning to heroin

In addition to learning about how, seemingly, inexpensive heroin was as compared to prescription opioids from individuals within respondents’ substance using social networks, respondents’ transition to heroin was also aided by patterns and shifts in their route of administration when using prescription opioids.

Eric began his opioid use with prescription pills that he started selling to supplement his regular income. Although he began taking the pills orally, he later learned that he could experience two distinct types of highs by sniffing one pill and taking another orally. Since the two routes of administration that are overwhelmingly associated with heroin use in the mid-Atlantic region are intranasal (through the nose) and intravenous (through the vein), Eric’s decision to begin sniffing his prescription opioids allowed for a seamless transition to snorting heroin.

Similarly, Brian noted that for the vast majority of his drug use period, he took prescription pills orally, only sniffing them once in a while. Sniffing was never his preference, but his willingness to consider it as a route of administration helped open the door for him to begin sniffing heroin. For Brian, it was the confluence of both a lesser cost and a willingness to change his regular route of administration that contributed to his transition from pills to heroin. In fact, of the 13 participants in this study who transitioned from prescription pills to heroin, nine began sniffing their pills prior to snorting heroin.

Mindy (32/Female) was also willing to sniff her prescription opioids on occasion, but once she was reunited with her best friend who had started using heroin, she too began sniffing heroin regularly.

***Interviewer:*** *When did you switch over from the pills to the dope?*

***Respondent:*** *I would say maybe, I was sniffing pills for like 3 maybe 4 years. Then I switched over… It was actually my best friend, and you know, we didn’t talk for like a couple months and then she came back around and she started doing dope around that time we weren’t talking for a few months, and then she came and you know, I sniffed it at first, and then went downhill after that. Pretty much.*

The respondents in this sample appear to have paved a smoother way for this transition from prescription opiates to heroin use to occur by creating a behavioral similarity between the two drugs through their route of administration. For individuals that had already shifted from taking prescription opioids orally to intranasally, purchasing heroin as a less expensive alternative was even easier because they had already adopted a common route of administration for heroin. The two respondents that did not transition to using heroin, also never used their prescription opioids any other way than orally.

#### Transitioning to intravenous use

None of the 15 respondents in this sample who initiated opioid use with prescription pills reported using pills intravenously. All of the individuals who used opioids intravenously in this sample (13) did so with heroin, including the respondents who had initiated their opioid use directly with heroin. Yet even for those individuals, it was members of personal social networks that presented the opportunity to initiate intravenous use. These informal mechanisms provided both information and influence.

For respondents like Tim (36/Male), this transition was as simple as being around a new girlfriend who used heroin intravenously. He was only able to capitalize on his openness to the experience once he formed a relationship with an individual who knew how to use and could facilitate his first time use. For respondents like Tami, all of her transition points came about due to influence exerted by, or information provided by, individuals she wound up spending time with at each point in her addiction. She explained her transition into shooting heroin this way:

***Respondent:*** *…Then my progression grew and grew and grew and that was that. So that’s how I came about with the heroin. Then, I got around a female, cause once you’re out there and around that stuff you’re with the wrong group of people and all that starts. So that girl, she shot, she did it intravenously, and she used to tell me it’s the best feeling and it will take your sickness immediately away and you’ll feel good, and da da da da da. So I started doing that, and that’s how the needle use began.*

It is important to note that this openness was not devoid of perceptions of risk. Tami was quite scared to use heroin intravenously, even believing that there was an imminent possibility she could overdose from a single use. This fear was shared by other respondents in the study, like Julie (32/Female), who didn’t use heroin until she was talked into it by someone very close to her:

***Respondent:*** *Yea, because I mean me and my husband had just taken Percocets, and one day we couldn’t get any but he know where [heroin] was, and he was like it’s just like a Perc and it’s cheaper. First I was like “No! I’m gonna die!”, and this and that. “I’m not shooting up!” Then he said, “no, you’re not going to overdose, it feels just like a Perc”. And I was like, “why? Have you done it?”, and he was like, “yea, I’ve tried it a couple times.” And I was like, “fine, let’s just try it.” So that’s how that started… But I mean, also because pretty much to even get in here [treatment program], like the shooters get more help and quicker than the sniffers do. So, like you at least have to have a track mark, so I’m like I might as well just try it, like there’s no other way.*

It is not surprising that these same informal networks also supplied information and advice about treatment options. By the time Julie transitioned to shooting heroin, other opioid users in her social network had informed her about the priority intake policies at several treatment clinics in the area. While this was not the sole reason for Julie’s change in route of administration, the fact that intravenous users are accepted for intake into the clinics more quickly contributed to her rationalization that shooting heroin would be okay, and perhaps even useful.

Other respondents actually used the same cost-benefit reasoning that led them from prescription opioids to heroin, in their decision to move from sniffing heroin to shooting heroin. Eric, began using heroin because it was a cheaper alternative to his prescription opioids, but noted that his tolerance increased at a much faster rate once he transitioned to heroin. His rationale for transitioning from sniffing heroin to shooting heroin was also a way to save money, because shooting heroin is a more efficient route of administration with a greater bioavailability.

Unfortunately for Eric, his life started unraveling at a much faster pace after his transition to heroin, and his switch to intravenous administration. He eventually lost his job, apartment, and custody of his two sons; he served 1 year in prison for theft and is now a convicted felon. Eric had never used an opioid before September 2012; by January 2013, he had transitioned to heroin. He entered his first detox facility in May 2013 and was incarcerated by April 2014.

#### Negative effects of intravenous heroin use

Eric was not the only patient that noted experiencing significant, and rapid, negative effects to his life from using heroin intravenously. Julie had a particularly poignant dialogue during her interview, where she reflected with utter disbelief about how quickly the transition from pills to heroin, and from sniffing to shooting, occurred.

***Respondent:*** *It was definitely, I’m still kinda shocked. Like wow, 6–7 months? And like all these years of taking Percs and like, I could go for a whole month taking them every day and just be fine. And I just never really thought that I was going to get addicted that FAST, and then to have the withdrawal symptoms like that, and you really do have to get up and it’s hard, to just stop. The way it completely takes over you, I’m still kind of like wow, how did this happen?? Like me and my husband, like especially the couple weeks prior to getting help, we’ve been talking about how we have to get help. And you know this whole first week together, you know getting help, like we even say, can you IMAGINE people who have been doing it for years? Like if we felt like we’re feeling, you know? And it’s only been like 6–7 months. Can you imagine? Like it’s already taken a toll on us, and our lives.*

Overall, respondents in this study maintained dependence on a fairly consistent level of prescription pills for a majority of their use periods. The 13 respondents in this study that initiated opioid use with prescription pills and later transitioned to heroin had an average of 9.2 years of opioid use in their lifetime – but only an average of 2.1 years of heroin use after making the transition. The most consistent reason respondents gave for transitioning from prescription pills to heroin was that they believed “it was *the same thing* as pills.” Besides the (presumed) difference in cost, users would have no reason to transition to heroin unless they believed pills and heroin where interchangeable. According to the respondents in this study, the notion of *interchangeability* is perhaps the most critical piece of misinformation being exchanged within substance-using networks. When asked how she would compare withdrawal experiences from prescription opioids and from heroin, Tami explained:

***Respondent:*** *From the heroin? How can I compare it? The withdrawal from the heroin and intravenous use is WORSE. It is deathly, deathly worse. I swear it is… There’s also a difference between the withdrawal from sniffing and a withdrawal from intravenous. There’s two different withdrawals from that too… It is the same set, but it’s more intense, and it comes on stronger. Like, I can wake up and be instantly sick as soon as I got out of bed. I’d be throwing up and using the bathroom in my pants, and all of that. Compared to if I sniffed, I would have a few hours before I got like that. Or a day even. But intravenous use, because it goes right into your vein it brings it on stronger, the withdrawal is worse.*

## Discussion

Over the course of the previous decade, existing literature has identified prescription opioid misuse and alternate forms of route of administration as potential precursors to future heroin use [6–17]. Among the most cited reasons for transitioning from prescription opioids to heroin are reduced cost and greater availability [6, 10, 16, 17]. This study sought to generate a nuanced understanding of this transition process how changes in route of administration might be related to participants’ transitioning from prescription opioids to heroin. Of utmost importance from these narratives was the notion of interchangeability between prescription opiates and heroin.

Consistent with previous qualitative analyses [13, 16], the participants in this study generally initiated their prescription opioid use with oral or intranasal administrations, followed by non-injection use of heroin, and finally, injection use of heroin. None of the participants in this study injected prescription opioids prior to initiating heroin use, which also corresponds to existing literature in this area [13, 16], but what is also worth noting, is that the participants in this study who never used prescription opioids any other way but orally, were the only ones who did not transition to heroin. These trends in route of administration and prescription opioid to heroin transitions indicate that behavioral treatment interventions should begin to consider that *how* participants use opioids may be just as important as *what* opioids they report using. This is especially important to consider beyond the normative emphasis placed on injection drug use, given its association with other public health risk factors such as contraction of HIV or Hepatitis C.

In addition to developing a greater understanding of the role of route of administration in prescription opioid to heroin transitions, this study also used patient narratives to debunk existing myths that have been promulgated widely in opioid use networks. Findings from this study expand on the literature in this area, which largely agrees that participants become convinced that heroin is the ‘same’ as prescription opioids, while also being cheaper and more easily available [10]. In fact, Cicero and colleagues [10] found that in their sample (*n* = 54) of participants who had reported previous or current use of prescription opioids, but at the time of participating had a primary drug of heroin, 94% reported using heroin because prescription opioids were more expensive and harder to obtain. While this study initially found respondents discussing similar reasons for transitioning to heroin, when asked to reflect upon if those reasons remained true throughout their heroin use, respondents indicated that they were false in the long run.

Other qualitative studies have focused on the route of administration transitions of youths with opioid use disorders, finding that this group largely attributed their transition to injection use to individual tolerance, cost, and shifting drug markets – findings that are consistent with those presented in this study [19]. Additionally, quantitative studies on this population found that lifetime prescription opioid dependence, early ago of prescription opioid initiation, use not attributed to self-medication of a health issue, and predominantly using prescription opioids non-orally were all significant predictors of youths transitioning to heroin [20]. By understanding the key role of route of administration and common myths shape key transition points for opioid users, practitioners will be allowed to develop effective harm reduction and prevention materials that target individuals already using prescription opioids, or in the early stages of heroin use. While myths surrounding the similarity of prescription opioids and heroin have been promulgated throughout opioid using networks, so too can these myths be debunked through successful and targeted information campaign strategies.

This study had several limitations. First, the data were collected from a purposive sample of participants who were receiving buprenorphine in a treatment clinic setting that primarily dispensed methadone. There are several pharmacotherapy-based settings in which individuals with opioid use disorder can access treatment, as well as many non-medication centered programs that offer abstinence-based treatment. It is possible that these buprenorphine respondents do not represent the experiences of participants entering other types of treatment for opioid use disorder. Second, there are many reasons individuals decide to access treatment at a given time (financial ability, court and family pressure, etc.), which makes this population selective, given that all of the participants were recruited into the study during their most recent treatment experience. Third, qualitative data collection centered around retrospective lines of inquiry may be subject to recall bias since some of the events and situations that were discussed may have occurred years prior to the interview.

## Conclusion

With the increased public attention on opioid use disorders, as well as concern surrounding a dramatic rise in opioid-related overdose deaths in recent years, understanding the association between prescription opioid use and transitioning to heroin has been at the forefront of opioid-related research. This study contributes two major findings to the existing body of literature in this area, namely: 1) a specific and nuanced understanding of *how* route of administration and transitions from prescription opioids to heroin occur, and 2) how myths surrounding the perceived benefits of transitioning from prescription opioids to heroin are debunked over time as individuals continue using heroin. Although preliminary, both of these findings point to the importance of generating harm reduction interventions that target individuals who may be at risk of transitioning, or have recently transitioned, to heroin.
